# Risk Factors, Treatments, and Outcomes of Adults Aged <55 Years With Acute Ischemic Stroke With Undetermined Versus Determined Pathogenesis: A Nationwide Swiss Cohort Study

**DOI:** 10.1161/JAHA.124.036761

**Published:** 2024-11-27

**Authors:** Tolga D. Dittrich, Thomas Schneider, Mira Katan, Andreas R. Luft, Marie‐Luise Mono, Manuel Bolognese, Krassen Nedeltchev, Timo Kahles, Marcel Arnold, Mirjam Heldner, Patrik Michel, Emmanuel Carrera, Biljana Rodic, Carlo W. Cereda, Nils Peters, Leo H. Bonati, Susanne Renaud, Andrea M. Humm, Friedrich Medlin, Sylvan Albert, Rolf Sturzenegger, Alexander A. Tarnutzer, Philip Siebel, Markus Baumgärtner, Christian Berger, Pasquale Mordasini, Jochen Vehoff, Gian Marco De Marchis, the Swiss Stroke Registry Investigators

**Affiliations:** ^1^ Department of Neurology University Teaching and Research Hospital St. Gallen St. Gallen Switzerland; ^2^ Department of Clinical Research University of Basel Basel Switzerland; ^3^ Department of Neurology University Hospital Basel and University of Basel Basel Switzerland; ^4^ Department of Neurology University Hospital Zurich Zurich Switzerland; ^5^ Department of Neurology Municipal Hospital Waid und Triemli Zurich Switzerland; ^6^ Department of Neurology Cantonal Hospital of Lucerne Lucerne Switzerland; ^7^ Department of Neurology Cantonal Hospital Aarau Aarau Switzerland; ^8^ Department of Neurology University Hospital Bern and University of Bern Bern Switzerland; ^9^ Department of Neurology University Hospital Lausanne Lausanne Switzerland; ^10^ Department of Neurology University Hospital Geneva Geneva Switzerland; ^11^ Department of Neurology Cantonal Hospital Winterthur Winterthur Switzerland; ^12^ Department of Neurology EOC Neurocenter of Southern Switzerland Lugano Switzerland; ^13^ Department of Neurology Hirslanden Hospital Zurich Zurich Switzerland; ^14^ Neurorehabilitation Rheinfelden Rehabilitation Clinic Rheinfelden Switzerland; ^15^ Department of Neurology Cantonal Hospital Neuchâtel Neuchâtel Switzerland; ^16^ Department of Internal Medicine, Neurological Unit HFR Fribourg – Cantonal Hospital Fribourg Switzerland; ^17^ Department of Neurology Cantonal Hospital Graubünden Chur Switzerland; ^18^ Department of Neurology Cantonal Hospital of Baden Baden Switzerland; ^19^ Department of Neurology Cantonal Hospital Frauenfeld Frauenfeld Switzerland; ^20^ Department of Neurology Cantonal Hospital Münsterlingen Münsterlingen Switzerland; ^21^ Department of Neurology Cantonal Hospital Grabs Grabs Switzerland; ^22^ Department of Neuroradiology Cantonal Hospital Aarau Aarau Switzerland

**Keywords:** ischemic stroke, undetermined pathogenesis, vascular risk factors, young, Cerebrovascular Disease/Stroke

## Abstract

**Background:**

The rising prevalence of acute ischemic stroke (AIS) in young adults, particularly with undetermined pathogenesis, is a growing concern. This study assessed risk factors, treatments, and outcomes between young AIS patients with undetermined and determined pathogeneses.

**Methods and Results:**

This was a retrospective cohort study including AIS patients aged 18 to 55 years in Switzerland, treated between 2014 and 2022. Stroke pathogeneses were classified using a modified TOAST (Trial of ORG 10172 in Acute Stroke Treatment) classification, with undetermined pathogenesis defined as no identified cause (including patent foramen ovale and cervical artery dissection). We examined vascular risk factors, acute treatments, 3‐month functional outcomes, and AIS recurrence within 3 months using logistic regression and Fine–Gray proportional hazards models. Of 3995 patients, 863 (22%) had undetermined pathogenesis. Compared with patients with determined pathogenesis, those with undetermined pathogenesis had a higher prevalence of dyslipidemia (54% versus 59%, *P*=0.007) and smoking (37% versus 43%, *P*=0.001), and were more likely to receive intravenous thrombolysis (27% versus 31%, *P*=0.046). Despite higher 3‐month AIS recurrence risk for the undetermined group (adjusted hazard ratio, 1.72 [95% CI, 1.01–2.94]), favorable functional outcomes at 3 months were more frequent (modified Rankin Scale score, 0–2: 90% versus 87%, *P*=0.033). Patients aged 46 to 55 years with undetermined pathogenesis had better outcomes than those with determined pathogenesis (modified Rankin Scale score, 0–1: 70% versus 64%, *P*=0.013; modified Rankin Scale score, 0–2: 89% versus 85%, *P*=0.023), while those aged 18 to 45 years showed higher recurrence rates (4.5% versus 1.8%, *P*<0.05) but similar functional outcomes.

**Conclusions:**

Young adults with AIS exhibit a considerable vascular risk burden. Those with undetermined pathogenesis displayed age‐related outcome disparities, with better short‐term outcomes in older and higher recurrence rates in younger patients.

**Nonstandard Abbreviations and Acronyms**
AIS: acute ischemic stroke
IAT: intra‐arterial treatment
IVT: intravenous thrombolysis
mRS: modified Rankin Scale
NAVIGATE ESUS: New Approach Rivaroxaban Inhibition of Factor Xa in a Global Trial Versus ASA to Prevent Embolism in Embolic Stroke of Undetermined Source
PFO: patent foramen ovale
RE‐SPECT ESUS: Randomized, Double‐Blind, Evaluation in Secondary Stroke Prevention Comparing the Efficacy and Safety of the Oral Thrombin Inhibitor Dabigatran Etexilate Versus Acetylsalicylic Acid in Patients With Embolic Stroke of Undetermined Source
SSR: Swiss Stroke Registry
SYSS: Swiss Young Stroke Study
TOAST: Trial of ORG 10172 in Acute Stroke Treatment

**Clinical Perspective**

**What Is New?**

- Young adults with acute ischemic stroke of undetermined pathogenesis have a high vascular risk burden, with elevated recurrence risk particularly in those aged 18 to 45 years, but show better functional outcomes, especially in those aged 46 to 55 years.

**What Are the Clinical Implications?**

- Improved targeted secondary prevention strategies are needed, particularly for young patients with AIS with undetermined pathogenesis aged <45 years, to address their elevated recurrence risk.

Stroke in young adults not only represents a growing concern due to its increasing incidence but also poses substantial socioeconomic implications, affecting individuals in their most productive years.^1^ Recent studies indicate that 10% to 15% of strokes occur in individuals aged <50 years, a demographic not traditionally considered at high stroke risk.^1^ Despite a general decline in stroke prevalence in high‐income countries, stroke incidence is rising among younger populations.^2^, ^3^

This trend has public health significance, considering the impact of stroke on the quality of life of young patients and their caregivers.^4^, ^5^ Approximately one third of young stroke survivors experience disabilities impacting daily activities and work participation,^6^, ^7^ with a 20‐year mortality rate of 26.8%, highlighting the urgent need for dedicated research focused on the management of modifiable vascular risk factors and pathogenesis‐tailored secondary prevention strategies.^8^

The observed increase in stroke incidence among young adults is partially attributed to an increasing prevalence of traditional vascular risk factors, such as smoking, hypertension, and dyslipidemia.^9^ While diabetes, hypertension, and obesity are prevalent and often undertreated in patients aged <55 years with stroke,^9^ these factors alone do not fully account for the rising stroke incidence in this age group, especially given the declining rates of myocardial infarction among the young. Recent studies indicate a rising number of ischemic cerebrovascular events among patients aged <55 years without established vascular risk factors, particularly in cryptogenic cases.^10^

Research on young adults (aged 18–49 years) with first‐ever acute ischemic stroke (AIS) revealed that traditional vascular risk factors are less prevalent in cryptogenic AIS than in cases with determined pathogenesis,^1^ suggesting a unique risk profile for this group. The difference in prognosis between determined and undetermined stroke pathogenesis cases, particularly in terms of functional outcome and recurrence rates in young patients, remains unclear. One study reported a higher functional independence rate at 3 months in patients with undetermined pathogenesis compared with those with a determined pathogenesis, without stratification by age group (79.7% versus 73.1%).^11^

In a Swiss cohort of young adults aged 18–55 years with AIS, we aimed to provide a description of the (1) prevalence of vascular risk factors, (2) acute treatments, (3) 3‐month functional outcomes, and (4) stroke recurrence risk and compare these aspects between patients with undetermined and determined pathogenesis.

## Methods

### Protocol Approval, Registration, and Data Availability

In accordance with Swiss legislation, enrollment in the SSR (Swiss Stroke Registry) is mandated for quality assurance measures. Patients are notified about the potential use for research with the provision that individuals who dissent to the use of their data are excluded from research projects. This study has received prior approval from the Ethics Committee Northwestern Switzerland (ID 2023–01248). Anonymized data and statistical code are available upon request.

### Study Design and Participants

This retrospective multicenter study used data from the SSR, a national, prospective registry designed for quality assurance and multicentric research in acute stroke care.

We included all consecutive patients aged 18–55 years with first‐ever, imaging‐confirmed AIS (ie, diffusion restriction on magnetic resonance imaging or arterial occlusion on computed tomography or magnetic resonance angiography or perfusion deficit on computed tomography/magnetic resonance perfusion imaging) treated at a certified Swiss stroke center or unit between January 2014 and September 2022. We excluded patients with incomplete diagnostic workup (defined as requiring at a minimum: extra‐ and intracranial assessment of cerebral arteries, ECG, and evaluation by a trained cardiologist), stroke mimics and those who declined research use of their data.

A detailed list of all baseline and follow‐up variables extracted from the SSR is provided in Data S1.

### Stroke Pathogenesis

The SSR uses a modified version of the TOAST (Trial of ORG 10172 in Acute Stroke Treatment) classification, categorizing AIS into 5 distinct categories: large‐artery atherosclerosis (≥50% stenosis), cardioembolic (excluding patent foramen ovale [PFO] or other rare cardiac sources), small‐vessel disease, stroke of other determined pathogenesis, and stroke of undetermined pathogenesis (despite complete diagnostic workup). In addition to these TOAST categories, the SSR captures specific causes like carotid artery dissection, PFO as the sole cause (with <3 vascular risk factors), and cases with >1 possible pathogenesis.

Further measures to improve data integrity are described in Data S2.

### Vascular Risk Factors and Manifest Vascular Disease

The SSR documents the following established vascular risk factors: arterial hypertension (defined as blood pressure >140/90 mm Hg or a history of hypertension), hyperlipidemia (indicated by lipid‐lowering therapy on admission or low‐density lipoprotein cholesterol >2.6 mmol/L), diabetes (excluding glucose intolerance), smoking (actively smoking or stopped <2 years ago), and body mass index upon admission. Additionally, it captures histories of transient ischemic attack, intracranial hemorrhage, coronary artery disease, presence of prosthetic heart valves, and low cardiac ejection fraction (<35%).

### Acute Treatment and Time Metrics

The following acute treatment–related information is recorded in the SSR: antiplatelet therapy (either single or dual), anticoagulants (oral or parenteral at therapeutic doses), intravenous thrombolysis (IVT) with recombinant tissue‐type plasminogen activator, onset‐to‐IVT time, door‐to‐IVT time, intra‐arterial treatment (IAT), onset‐to‐IAT time, and door‐to‐IAT time.

### Functional Outcome and Stroke Recurrence

Functional outcome measures recorded in the SSR include the modified Rankin Scale (mRS) score at 3 months. Method of follow‐up (ranging from in‐person clinic visits and telephone interviews to medical record reviews) is chosen by each SSR participating center. Recurrent strokes and deaths are systematically captured throughout the 3‐month follow‐up period.

### Outcome Measures

Outcome measures were the prevalence of traditional vascular risk factors (arterial hypertension, diabetes, dyslipidemia, smoking), the proportion receiving acute treatments (IVT, IAT, antiplatelet treatment, and therapeutic anticoagulation), functional outcome at 3 months measured by the mRS score (ordinal and dichotomized [0–1 versus 2–6; 0–2 versus 3–6]; with mRS scores of 0–1 indicating excellent, and 0–2 favorable outcome), and AIS recurrence within 3 months.

### Statistical Analysis

Statistical analyses compared patients with undetermined stroke pathogenesis and (1) those with determined pathogeneses on the basis of the modified TOAST classification (large‐artery atherosclerosis, cardioembolic, small‐vessel occlusion, stroke of other determined pathogenesis, carotid artery dissection, PFO, >1 possible pathogenesis), and (2) an aggregated group including all patients with determined stroke pathogenesis. Additionally, we compared patients with undetermined pathogenesis to those with determined pathogenesis within the age subgroups of 18 to 45 and 46 to 55 years.

Categorical variables are presented as counts with percentages, and continuous variables as medians with interquartile ranges (IQRs). The Kruskal–Wallis rank‐sum test (continuous variables) and Pearson χ^2^ test (categorical variables) were used to assess group differences.

We fitted logistic regression models to predict good (mRS score, 0–2 versus 3–6) and excellent functional outcomes (mRS score, 0–1 versus 2–6) at 3 months follow‐up using maximum likelihood estimation. To assess whether an undetermined stroke pathogenesis independently affects functional outcome, we fitted models that included age, sex, pathogenesis (determined versus undetermined), and interactions between age and pathogenesis, and sex and pathogenesis. Sensitivity analyses involved fitting separate models for age subgroups (18–45 and 46–55 years). Additionally, we fitted models using the modified TOAST stroke pathogenesis (as a factor) with undetermined pathogenesis as the reference, controlling for age (centered at the grand population mean) and sex (female as reference).

Fine–Gray proportional hazards models, adjusted for age and sex, evaluated the impact of undetermined (versus determined) stroke pathogenesis on stroke recurrence within 3 months, considering death as a competing risk.^12^ Event of recurrent stroke and death were censored at 3 months for cumulative incidence and proportional hazards analyses. Analyses were limited to cases with complete covariable sets, and model terms were assessed using the Wald test. This analysis was conducted for the total data set as well as for the age subgroups 18 to 45 and 46 to 55 years.

Missing values were discarded from analyses. The number of observations is given for all variables and models in the Table 1 and Table S1, respectively. *P* values <0.05 were considered significant, and we report 95% CIs.

**Table 1:** Patient Characteristics by Stroke Pathogenesis

Characteristic	No.	Overall, n=3995*	Undetermined pathogenesis, n=863*	Cervical artery dissection, n=631*	PFO, n=607*	Cardiac embolism, n=547*	Other determined pathogenesis, n=382*	Small‐vessel disease, n=370*	Large‐artery atherosclerosis, n=370*	>1 possible pathogenesis, n=225*	*P* value†
Age	3995	48.2 (41.5–52.1)	48.6 (42.9–52.4)	44.7 (38.2–50.4)	44.9 (36.5–50.4)	49.5 (43.9–52.7)	47.2 (38.9–51.4)	50.3 (46.5–52.9)	51.3 (47.5–53.6)	49.7 (43.8–52.5)	<0.001
Male sex	3992	2627 (66)	547 (63)	422 (67)	372 (61)	375 (69)	212 (55)	278 (75)	273 (74)	148 (66)	<0.001
Body mass index	3202	25.7 (23.1–29.3)	26.2 (23.5, 29.7)	24.7 (22.5–27.7)	24.7 (22.1–28.0)	26.3 (23.7, 30.6)	25.4 (22.3–28.2)	26.8 (24.2–31.3)	26.5 (24.2–30.1)	25.8 (23.1–28.7)	<0.001
Hypertension	3995	1485 (37)	316 (37)	169 (27)	73 (12)	229 (42)	114 (30)	275 (74)	222 (60)	87 (39)	<0.001
Hyperlipidemia	3988	2206 (55)	512 (59)	263 (42)	273 (45)	296 (54)	158 (41)	294 (79)	273 (74)	137 (61)	<0.001
Diabetes	3995	416 (10)	75 (8.7)	26 (4.1)	13 (2.1)	65 (12)	25 (6.5)	88 (24)	94 (25)	30 (13)	<0.001
Smoking	3987	1522 (38)	370 (43)	145 (23)	177 (29)	204 (37)	140 (37)	187 (51)	204 (55)	95 (42)	<0.001
Number of vascular risk factors	3995	1.0 (1.0–2.0)	2.0 (1.0–2.0)	1.0 (0.0–2.0)	1.0 (0.0, 2.0)	2.0 (1.0–3.0)	1.0 (0.0–2.0)	2.0 (2.0, 3.0)	2.0 (2.0–3.0)	2.0 (1.0–2.0)	<0.001
Atrial fibrillation/flutter	3995	182 (4.6)	0 (0)	3 (0.5)	3 (0.5)	155 (28)	7 (1.8)	4 (1.1)	3 (0.8)	7 (3.1)	<0.001
Coronary heart disease	3988	188 (4.7)	30 (3.5)	6 (1.0)	3 (0.5)	84 (15)	16 (4.2)	13 (3.5)	21 (5.7)	15 (6.7)	<0.001
Prosthetic valves	3990										<0.001
None		3901 (98)	861 (100)	629 (100)	606 (100)	479 (88)	372 (98)	369 (100)	368 (100)	217 (96)	
Mechanical		68 (1.7)	0 (0)	1 (0.2)	0 (0)	58 (11)	4 (1.0)	1 (0.3)	0 (0)	4 (1.8)	
Biological		21 (0.5)	1 (0.1)	1 (0.2)	0 (0)	10 (1.8)	5 (1.3)	0 (0)	0 (0)	4 (1.8)	
Ejection fraction (<35%)	3118	72 (2.3)	3 (0.4)	3 (0.6)	2 (0.4)	57 (14)	3 (1.0)	1 (0.4)	1 (0.4)	2 (1.1)	<0.001
Prior transient ischemic attack	3995	110 (2.8)	20 (2.3)	16 (2.5)	9 (1.5)	13 (2.4)	13 (3.4)	11 (3.0)	23 (6.2)	5 (2.2)	0.002
Prior intracerebral hemorrhage	3994	31 (0.8)	11 (1.3)	1 (0.2)	0 (0%)	5 (0.9)	7 (1.8)	1 (0.3)	3 (0.8)	3 (1.3)	0.010
Total cholesterol, mmol/L	3187	4.9 (4.1 5.7)	5.0 (4.2–5.7)	4.7 (3.9–5.4)	4.5 (3.9–5.2)	4.7 (3.9–5.7)	4.5 (3.8–5.4)	5.3 (4.6–6.0)	5.4 (4.5–6.3)	4.9 (4.0–5.8)	<0.001
Low‐density lipoprotein cholesterol, mmol/L	3144	3.0 (2.3–3.7)	3.1 (2.4–3.7)	2.8 (2.2–3.4)	2.7 (2.1–3.3)	2.9 (2.2–3.8)	2.7 (2.1–3.4)	3.4 (2.8–4.1)	3.5 (2.6–4.3)	3.1 (2.3–3.9)	<0.001
Initial National Institutes of Health Stroke Scale score	3951	2.0 (1.0–7.0)	2.0 (1.0–6.0)	2.0 (0.0–8.0)	2.0 (0.0, 4.0)	4.0 (1.0–9.0)	3.0 (1.0–9.0)	2.0 (1.0–4.0)	3.0 (1.0–10.0)	3.0 (1.0–6.0)	<0.001
Transthoracic echocardiogram	3559	2384 (67)	551 (72)	289 (52)	366 (68)	358 (71)	215 (63)	255 (76)	205 (64)	145 (71)	<0.001
Transesophageal echocardiogram	3521	1355 (38)	369 (48)	48 (8.7)	382 (72)	175 (35)	141 (41)	85 (26)	70 (22)	85 (43)	<0.001
Start antiplatelet therapy	3991	2205 (55)	514 (60)	348 (55)	316 (52)	201 (37)	177 (46)	288 (78)	225 (61)	136 (60)	<0.001
Start anticoagulants	3990	300 (7.5)	25 (2.9)	83 (13)	21 (3.5)	89 (16)	32 (8.4)	8 (2.2)	22 (5.9)	20 (8.9)	<0.001
Intravenous thrombolysis	3847	1073 (28)	256 (31)	161 (27)	215 (36)	165 (31)	74 (20)	60 (17)	99 (28)	43 (20)	<0.001
Intra‐arterial treatment	3849	769 (20)	152 (18)	156 (26)	95 (16)	143 (27)	85 (23)	3 (0.8)	95 (27)	40 (19)	<0.001
mRS score at follow‐up, 0–2 (vs 3–6)	3445	3009 (87)	671 (90)	463 (84)	513 (96)	398 (85)	261 (76)	282 (93)	253 (82)	168 (85)	<0.001
Recurrent stroke until follow‐up	3394	76 (2.2)	21 (2.8)	13 (2.4)	2 (0.4)	9 (2.0)	11 (3.4)	6 (2.0)	9 (3.0)	5 (2.6)	0.076

mRS indicates modified Rankin Scale; and PFO, patent foramen ovale.

* Median (interquartile range) or frequency (%).

^†^ Kruskal–Wallis rank‐sum test; Pearson's χ^2^ test.

All analyses were performed with RStudio version 4.3.0 (R Foundation for Statistical Computing, Vienna, Austria). Data S3 provides package details. Our reporting adheres to the Strengthening the Reporting of Observational studies in Epidemiology guidelines.^13^

## Results

**Figure 1:**
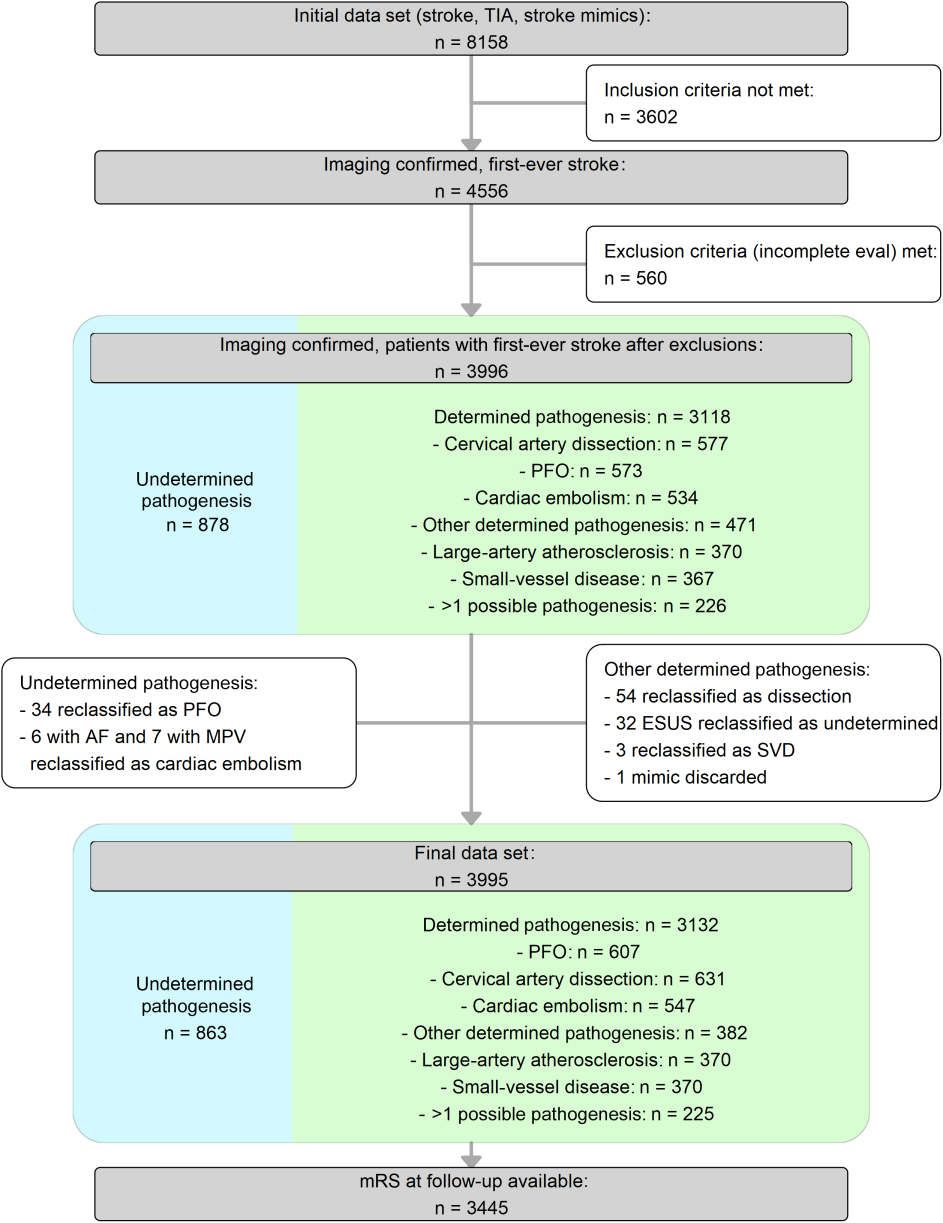
Patient flowchart. AF indicates atrial fibrillation; BPV, biological prosthetic valve; ESUS, embolic stroke of unknown source; LEF, low ejection fraction; MPV, mechanical prosthetic valve; mRS, modified Rankin Scale; PFO, patent foramen ovale; SVD, small‐vessel disease; and TIA, transient ischemic attack.

**Figure 2:**
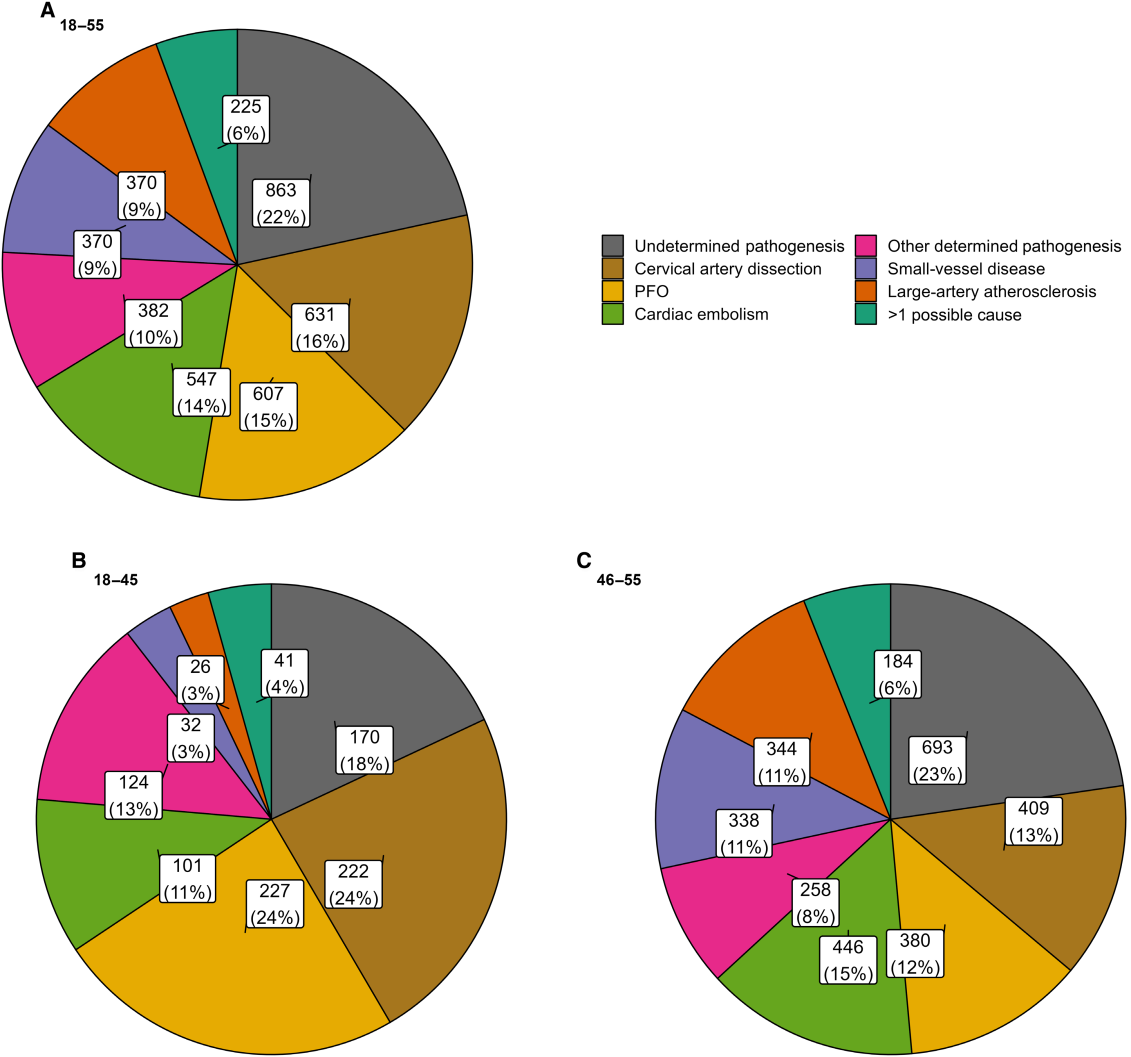
Distribution of stroke pathogenesis. (**A**), All patients; (**B**), patients aged 18–45 y, (**C**), patients aged 46–55 y. PFO indicates patent foramen ovale.

Among 8158 individuals aged 18 to 55 years with AIS, transient ischemic attack, or mimics, 4556 (60%) were identified with first‐ever AIS confirmed by imaging (Figure 1). After exclusions for incomplete diagnostics (n=560), 3996 patients (median age, 48.2 [IQR, 41.5–52.1] years; 66% male sex) were analyzed: 863 with undetermined pathogenesis and 3132 with determined pathogenesis (Figure 2). Three‐month follow‐up was available for 3445 patients (86.2%). Of 943 patients aged 18 to 45 years, 773 had determined pathogenesis and 170 undetermined. Follow‐up data were available for 818 (662 determined, 156 undetermined). Of 3052 patients aged 46 to 55 years, 2359 (77.3%) had determined pathogenesis and 693 undetermined. Follow‐up data were available for 2627 (2035 determined, 592 undetermined).

AIS with undetermined pathogenesis after complete diagnostic evaluation (863 cases [22%]) was more prevalent than any single determined pathogenesis (Table 1). Sex distribution was similar between patients with undetermined and determined stroke pathogenesis, with a higher proportion of men in the small‐vessel disease (75%) and large‐artery atherosclerosis groups (74%; Table S1). Determined pathogenesis cases included PFO (15%), cervical artery dissection (16%), and cardiac embolism (excluding PFO or other rare cardiac causes; 14%). Other determined pathogeneses comprised small‐vessel disease and large‐artery atherosclerosis (≥50% stenosis; each 9.3%), and multiple potential pathogeneses in 5.6% of cases. A total of 9.6% of patients had a stroke of other determined pathogenesis, with notable shifts in the distribution of determined pathogeneses between age groups 18 to 45 and 46–55 years (see Table S2).

### Vascular Risk Factors

**Figure 3:**
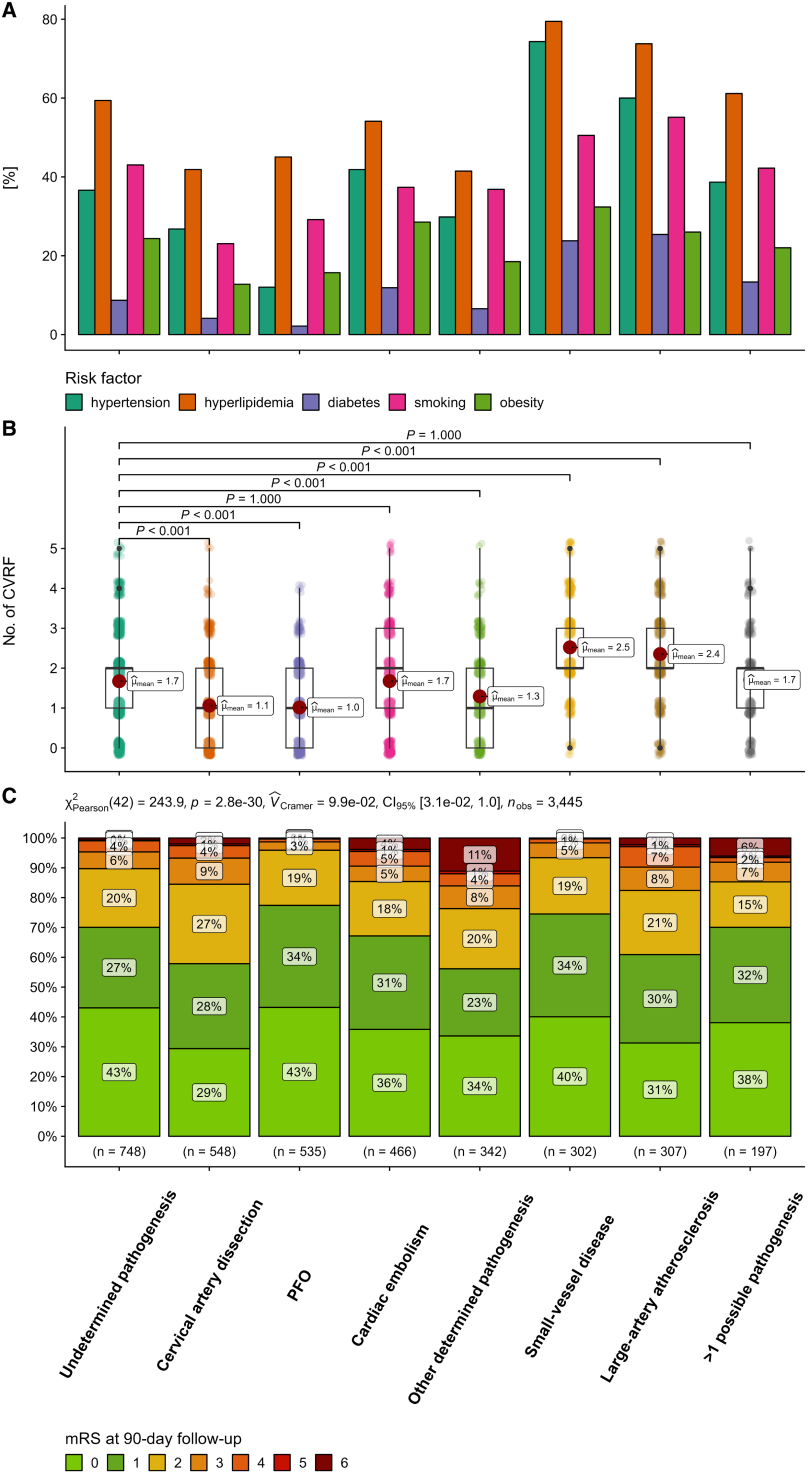
Vascular risk factors with hold‐adjusted Wilcoxon group comparisons of the number of vascular risk factors between patients with undetermined and specific determined pathogeneses (A, B) and distribution of mRS scores at 3 months (C) by stroke pathogenesis. mRS indicates modified Rankin Scale; and PFO, patent foramen ovale.

The most common risk factors were dyslipidemia (55%), smoking (38%), and arterial hypertension (37%). Analyses revealed differences in vascular risk profiles between patients with undetermined and determined pathogenesis. Those with undetermined pathogenesis had slightly higher body mass index compared with those with determined pathogeneses (median, 26.2 versus 25.7 kg/m^2^, *P*=0.004) and higher rates of dyslipidemia (59% versus 54%, *P*=0.007) and smoking (43% versus 37%, *P*=0.001). The proportion of patients with at least 1 vascular risk factor was higher in the undetermined group (83% versus 77%, *P*<0.001; Figure 3B). The risk profile of undetermined pathogenesis patients resembled those with cardiac embolism and multiple possible pathogeneses, with similar rates of smoking, arterial hypertension, diabetes, and dyslipidemia (Table 1 and Figure 3A). Patients with cardiac embolism had the highest incidence of atrial fibrillation (28%), coronary heart disease (15%), prosthetic valves (mechanical, 11%; biological, 1.8%), and low cardiac output (15%). Both follow‐up transthoracic echocardiogram (72% versus 66%, *P*=0.001) and transesophageal echocardiogram (48% versus 36%, *P*<0.001) were performed more frequently in patients with undetermined pathogenesis than in patients with determined pathogenesis.

In the age subgroup analyses, the number of vascular risk factors was higher in the undetermined compared with the determined pathogenesis group for patients aged 18 to 45 (median, 1 [IQR, 0–2] years versus median, 1 [IQR, 0–2]; *P*=0.011). No significant differences were found in the age group 46–55 years (see Table S2 and Figure S1).

### Stroke Severity and Acute Treatment

Initial National Institutes of Health Stroke Scale scores were generally low (median, 2 [IQR, 1–7]; Table 1), highest among cardiac embolism (median, 4 [IQR, 1–9]), large‐artery atherosclerosis (median, 3 [IQR, 1–10]), other determined pathogenesis (median, 3 [IQR, 1–9]), and multiple possible pathogenesis groups (median, 3 [IQR, 1–6]), without evident differences between undetermined and determined pathogeneses (median, 2 [IQR, 1–6] versus median, 2 [IQR, 1–7]; *P*>0.9).

IVT was administered in 28% of cases, more frequently in patients with undetermined pathogenesis compared to those with determined pathogeneses (31% versus 27%, *P*=0.046), with the highest usage in patients with PFO (36%) and the lowest in small‐vessel disease and other determined pathogenesis groups (17% and 20%, respectively). IAT was performed in 20% of cases, with the highest rates observed in patients with large‐artery atherosclerosis, cardiac embolism (each 27%), and cervical artery dissection (26%) as compared with a rate of 18% in the undetermined pathogenesis group. In the age subgroup analyses, no significant differences in IVT and IAT rates were observed between patients with undetermined and determined pathogenesis.

### Functional Outcomes

Excellent functional outcome (mRS score, 0–1) at 3 months was achieved by 67%, and favorable (mRS score, 0–2) by 87% (Table 1). Strokes of undetermined pathogenesis had higher rates of favorable outcomes compared with those with determined origin (90% versus 87%, *P*=0.033; Figure 3C and Table S1). This difference was particularly evident in the 46 to 55 years age group, where the undetermined pathogenesis group had higher rates of both excellent and favorable outcomes compared with the determined pathogenesis group (mRS score, 0–1: 70% versus 64%, *P*=0.013; mRS score, 0–2: 89% versus 85%, *P*=0.023; Table S2). In contrast, there were no significant differences in outcomes between the groups in the 18 to 45 years age group (mRS score, 0–1: 71% versus 72%, *P*=0.8; mRS score, 0–2: 93% versus 92%, *P*=0.7). The highest proportions of unfavorable outcomes (mRS score, 3–6) were seen in patients with other determined pathogenesis (24%).

Logistic regression analyses revealed that the odds of favorable outcomes (mRS score, 0–2; see Table S3) decreased with age (adjusted odds ratio [aOR], 0.95 [95% CI, 0.93–0.96]), while there was a positive interaction effect between age and undetermined pathogenesis (aOR, 1.04 [95% CI, 1.00–1.08]). The main effect of undetermined pathogenesis, male sex, and the interaction between age and sex were not significant predictors of favorable outcomes. In the sensitivity analysis for the 18 to 45 years age subgroup (Table S4), only age remained a significant predictor of lower odds of favorable outcomes (aOR, 0.95 [95% CI, 0.89–1.00]), with other factors not reaching statistical significance. In the 46 to 55 years age subgroup (Table S5), the odds of favorable outcomes decreased with age (aOR, 0.92 [95% CI, 0.89–0.95]), while a stronger positive interaction effect between age and undetermined pathogenesis was observed (aOR, 1.15 [95% CI, 1.07–1.28]; for excellent functional outcomes see Table S6–S8). The main effects of undetermined pathogenesis, male sex, and the age–sex interaction remained nonsignificant in this subgroup.

The logistic regression model including the modified TOAST classification revealed that PFO (aOR, 2.35 [95% CI, 1.46–3.92]; Table S9 and Figure S2) and small‐vessel disease (aOR, 1.78 [95% CI, 1.08–3.04]) had higher odds for favorable outcomes (mRS score, 0–2) compared with undetermined pathogeneses, whereas other determined stroke pathogeneses (aOR, 0.34 [95% CI, 0.24–0.49]), cervical artery dissection (aOR, 0.55 [95% CI, 0.39–0.77]), large‐artery atherosclerosis (aOR, 0.61 [95% CI, 0.42–0.90]), and cardiac embolism (aOR, 0.71 [95% CI, 0.50–1.01]) were associated with lower odds (for excellent outcomes [mRS score, 0–1] see Figure S3).

For excellent outcomes (mRS score, 0–1; Tables S6 and S10, and Figure S2), age was associated with lower odds (aOR, 0.97 [95% CI, 0.96–0.98]). There was a positive interaction effect between age and undetermined pathogenesis (aOR, 1.03 [95% CI, 1.00–1.05), but no significant main effect of undetermined stroke pathogenesis. In the sensitivity analysis for the age 18 to 45 years subgroup (Table S7), both the negative main effect of age (aOR, 0.94 [95% CI, 0.91–0.97]) and the positive interaction effect between age and undetermined pathogenesis (aOR, 1.08 [95% CI, 1.01–1.16]) remained significant. In contrast, no significant effects were observed in the age 46 to 55 years subgroup (Table S8). PFO‐related strokes were associated with higher odds (aOR, 1.36 [95% CI, 1.05–1.76]; Table S10), while other stroke pathogeneses (aOR, 0.53 [95% CI, 0.41–0.69]), cervical artery dissection (aOR, 0.55 [95% CI, 0.43–0.69), and large‐artery atherosclerosis (aOR, 0.71 [95% CI, 0.53–0.94]) had lower odds.

### Ischemic Stroke Recurrence Within 3 Months

**Figure 4:**
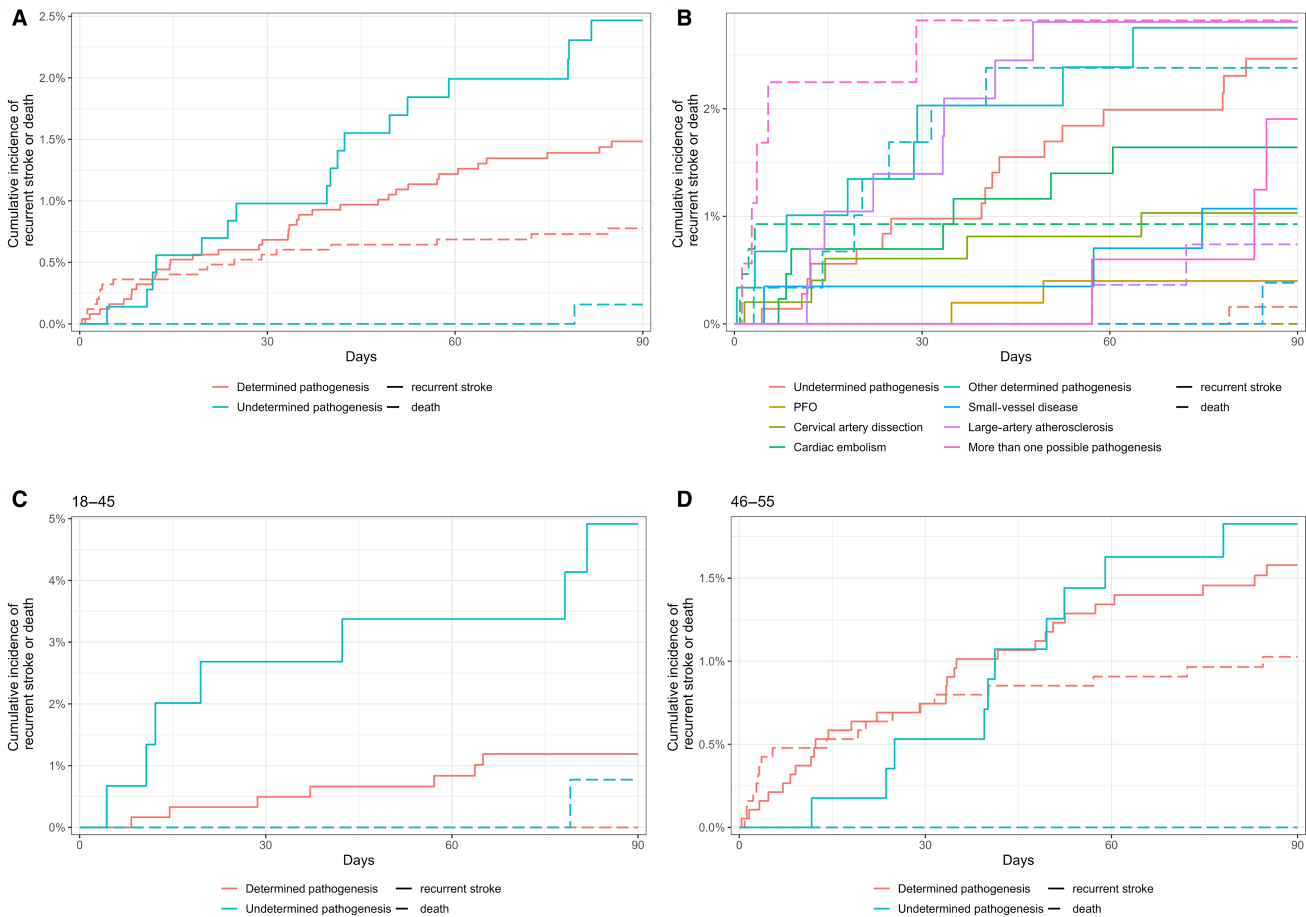
Cumulative incidence of stroke recurrence and death by stroke pathogenesis. **A**, All patients, dichotomized pathogeneses (undetermined vs determined); (**B**) all patients, modified TOAST pathogeneses; (**C**) patients aged 18–45 y, dichotomized pathogeneses; (**D**) patients aged 46–55 y, dichotomized pathogeneses. mRS indicates modified Rankin Scale; PFO, patent foramen ovale; and TOAST, Trial of ORG 10172 in Acute Stroke Treatment.

In the Fine–Gray proportional hazards models, patients with undetermined pathogenesis exhibited a higher risk of stroke recurrence within 3 months compared with those with determined pathogenesis (adjusted hazard ratio [aHR], 1.72 [95% CI, 1.01–2.94]; Table S11). Subgroup analyses by age revealed that this increased risk was particularly pronounced in the age 18 to 45 years group with undetermined pathogenesis (aHR, 3.42 [95% CI, 1.15–9.11]; Table S12). In contrast, there was no significant difference in stroke recurrence risk between undetermined and determined pathogeneses in the age 46–55 years group (aHR, 1.37 [95% CI, 0.73–2.57]; Table S13). Comparing undetermined pathogenesis with each specific stroke pathogenesis (Table S14), PFO presented a lower recurrence risk compared with undetermined pathogenesis (aHR, 0.12 [95% CI, 0.03–0.54]), while other pathogeneses showed no difference. Cumulative incidence graphs illustrate stroke recurrence and death by pathogenetic groups (Figure 4).

## Discussion

This study investigated the stroke pathogeneses in young adults aged 18 to 55 years across Switzerland, focusing on contrasting undetermined and determined causes. Our main findings are (1) traditional vascular risk factors are highly prevalent in both patients with undetermined and determined AIS pathogenesis; (2) rates of IVT and IAT were overall high in the studied population, with patients with undetermined pathogenesis being more likely to receive IVT; (3) patients with undetermined pathogenesis aged 46 to 55 years exhibited more favorable 3‐month functional outcomes compared with those with determined pathogenesis, while no significant differences were found in the age 18 to 45 years group; (4) AIS recurrence rates within 3 months were higher in patients with undetermined pathogenesis compared with those with determined pathogenesis, particularly in the age 18 to 45 years group; and (5) there were pronounced age disparities in patients with undetermined pathogenesis, with higher stroke recurrence rates in the age 18 to 45 years group and better functional outcomes in the age 46 to 55 years group compared with those with determined pathogenesis.

We found that ≈22% of AIS in young adults (aged 18–55 years) had undetermined pathogenesis, with a lower rate of 18% in patients aged 18 to 45 years. This corresponds closely to the 25.2% observed in a recent Dutch multicenter study.^1^ Vascular risk factors were prevalent among patients with AIS with dyslipidemia (55%), current smoking (38%), and arterial hypertension (37%) being the most common, showing a rise in dyslipidemia compared with the rates reported by Putaala et al,^14^ where dyslipidemia was 45.8%, current smoking 48.7%, and hypertension 35.9%. Patients with undetermined stroke pathogenesis exhibited higher body mass index and increased rates of dyslipidemia and smoking but remarkably a trend toward less diabetes than those with determined pathogeneses. While these vascular risk factors were less common in patients with undetermined pathogenesis compared with those with small‐vessel or large‐artery atherosclerosis strokes, which are known to be highly associated with these risk factors,^9^, ^14^ the similarity in risk profiles between undetermined pathogenesis and cardiac embolism may suggest potential hidden cardioembolic mechanisms underlying cryptogenic embolic stroke.^15^, ^16^ Overall, the number of vascular risk factors was higher in patients with undetermined pathogenesis compared with those with determined pathogenesis, particularly in the age 18–45 years group. The findings reinforce the results of a recent Dutch multicenter study involving 1322 patients with AIS aged 18 to 49 years, showing that traditional vascular risk factors were frequent in cryptogenic AIS.^1^

Regarding revascularization therapies, we observed high rates of IVT (28% versus 18.2%^1^) and IAT (20% versus 2.6%^1^), exceeding IVT rates in the UK and US general stroke population (11%–12%^17^, ^18^) and the European Stroke Action Plan's 15% target for 2018 to 2030.^19^ Notably, patients with undetermined pathogenesis had slightly higher IVT rates (31%) than the determined pathogenesis group (27%), particularly compared with patients with small‐vessel disease (17%). IAT rates were similar between undetermined (18%) and determined pathogeneses (20%), but higher in specific pathogeneses like cardiac embolism (27%), large‐artery atherosclerosis (27%), and cervical artery dissection (26%). The preference for antiplatelets over anticoagulation in cases with undetermined pathogenesis aligns with the NAVIGATE ESUS (New Approach Rivaroxaban Inhibition of Factor Xa in a Global Trial Versus ASA to Prevent Embolism in Embolic Stroke of Undetermined Source) and RE‐SPECT ESUS (Randomized, Double‐Blind, Evaluation in Secondary Stroke Prevention Comparing the Efficacy and Safety of the Oral Thrombin Inhibitor Dabigatran Etexilate Versus Acetylsalicylic Acid in Patients With Embolic Stroke of Undetermined Source) trial findings, which showed no benefit of oral anticoagulation over aspirin in cryptogenic embolic stroke.^20^, ^21^

Our third finding was that two thirds of young patients with AIS achieved excellent functional outcome (mRS score, 0–1) at 3 months, and 87% attained good functional outcomes (mRS score, 0–2), corresponding to the results from the SYSS (Swiss Young Stroke Study; young patients aged 16–55 years with AIS; 61% for mRS score, 0–1)^7^ and an older Swiss analysis (68% for mRS score, 0–1)^6^ without showing increased favorable outcomes with improved access to intra‐arterial interventions. The undetermined pathogenesis group did not achieve the highest favorable outcome rates seen in PFO (77%) and small‐vessel disease (75%), nor the lowest observed in other determined causes (56%) and cervical artery dissection (58%). Patients with undetermined pathogenesis had a 90% chance of good outcome, slightly higher than the 87% in the determined pathogenesis group. Outcome disparities across pathogeneses were evident, with PFO and small‐vessel disease being more likely, and those with other determined pathogeneses being less likely to achieve favorable functional outcomes. Outcome disparities were more pronounced in the age 46 to 55 years group (mRS score, 0–1: 70% versus 64%; mRS score, 0–2: 89% versus 85%), with no significant differences in the age 18–45 years group (mRS score, 0–1: 71% versus 72%; mRS score, 0–2: 93% versus 92%, *P*=0.7).

Significant interaction effects suggest that the odds of excellent and favorable outcomes decrease less with age in the undetermined pathogenesis group compared with the determined group, especially for excellent outcomes in ages 18 to 45 years and favorable outcomes in ages 46 to 55 years. This age disparity cannot be solely explained by differing proportions of determined stroke pathogeneses. While the proportion of large‐artery atherosclerosis and cardioembolic strokes (associated with worse outcomes) increased with age, the proportion of cervical artery dissection (also associated with worse outcomes) decreased, and small‐vessel disease strokes (associated with better outcomes) increased in the age 46 to 55 years group. Further investigation is needed to understand the reasons behind this age disparity.

AIS recurrence within 3 months was higher in the undetermined pathogenesis group (2.8%) than in the determined pathogenesis group (2.1%), a statistically significant difference when accounting for the competing risk of death. Pronounced age disparities were observed, with significantly higher AIS recurrence rates in the undetermined pathogenesis group (4.5%) versus the determined pathogenesis group (1.8%) among patients aged 18 to 45 years, as indicated by Fine–Gray proportional hazards models. No significant differences were found in the age 46 to 55 years group (2.4% versus 2.2%).

While a lower recurrence rate of stroke in patients with PFO partially explains the difference between undetermined and determined pathogenesis groups, further research is needed to explore the high rate of recurrent AIS within 3 months in patients aged 18 to 45 years with undetermined stroke pathogenesis.

The overall 2.8% 3‐month recurrence rate in patients with undetermined pathogenesis is higher than the 1.9 per 100 patient‐years in patients with embolic stroke of unknown source reported by Perera et al.^22^ and possibly exceeds Nedeltchev and colleagues’^6^ reported 3% annual risk across all patients. Long‐term data from 396 young patients with stroke showed a 10.6% recurrence over a median follow‐up period of 11.8 years.^23^ Notably, most patients who experienced recurrent strokes and were initially categorized under undetermined cause were later reclassified as large‐artery atherosclerosis, cardioembolism, small‐vessel occlusion, or other determined causes.^23^ These findings, along with a 15% long‐term (>10 years) AIS and transient ischemic attack recurrence rate in patients with embolic stroke of unknown source contrast the considerably lower 5.5% new incidence rate of atrial fibrillation and underscore the need for improved understanding of the pathophysiology of first‐ever and recurrent ischemic cerebrovascular events in these patients.^24^ The discrepancy between functional outcomes and stroke recurrence rates in the undetermined pathogenesis group may seem unexpected. This observation could stem from inherent adverse prognostic factors in this group or more likely, the absence of a specific drug target for secondary prevention and the group's residual heterogeneity including undetected rare stroke causes.

Notably, 9.6% of patients had other identified causes, experiencing the highest rate of unfavorable outcomes (mRS score, 3–6; 24%), short‐term stroke recurrence (3.4%), and death (3.4%), warranting further investigation of this heterogenous subgroup.

### Strengths and Limitations

Strengths include (1) by using data from all Swiss stroke centers contributing to the prospective SSR (participation in the registry is mandatory for certification), a large, national, comprehensive sample was obtained, reducing potential selection bias; and (2) the comprehensive elucidation of several relevant aspects including functional outcomes, with previous studies having often focused on only partial aspects.

Limitations include (1) the lack of further information on pathogenetic diagnostic workup performed within the group with undetermined pathogenesis (such as laboratory thrombophilia screenings or rare inflammatory and autoimmune causes); (2) the use of the TOAST classification to categorize pathogeneses, which does not provide information on the certainty of the classification; (3) the limited generalizability of the results to other countries and regions with different demographics or health care infrastructure.

## Conclusions

In adults aged 18–55 with AIS, vascular risk factors were prevalent across all pathogeneses, including those with undetermined pathogenesis. Despite higher AIS recurrence rates in these patients, they exhibited more favorable functional outcomes, emphasizing the need for tailored prevention strategies and further research.

## Sources of Funding

The SSR was financially supported by the Swiss Heart Foundation (the funder). The funder had no influence on the study design, data collection, or data analysis.

## Disclosures

None.

## Supporting information

Swiss Stroke Registry: List of Collaborators
Data S1–S3
Tables S1–S14
Figures S1–S3
