# Supplementary material for: Risk Factors, Treatments, and Outcomes of Adults Aged <55 Years With Acute Ischemic Stroke With Undetermined Versus Determined Pathogenesis: A Nationwide Swiss Cohort Study
Source: J Am Heart Assoc. 2024 Nov 27;13(23):e036761. doi: 10.1161/JAHA.124.036761 (PMC11681592; doi:10.1161/JAHA.124.036761)
Supplement: Supplementary file 1 — Swiss Stroke Registry: List of Collaborators Data S1–S3 Tables S1–S14 Figures S1–S3 [file JAH3-13-e036761-s001.pdf]

SUPPLEMENTAL MATERIAL

Swiss Stroke Registry: List of collaborators

Center	***Collaborators***
**Stroke Center (alphabetical order)**	
Cantonal Hospital Aarau (KSA)	Timo Kahles<br>Krassen Nedeltschev
University Hospital Basel (USB) and University of Basel	Valerian Altersberger<br>Kristin Blackham<br>Leo Bonati<br>Alex Brehm<br>Gilles Dulith<br>Amgad El Mekabaty<br>Stefan T. Engelter<br>Urs Fisch<br>Joachim Fladt<br>Hernik Gensicke<br>Lisa Hert<br>Philippe Lyrer<br>Sabrina Manuzzi<br>Marina Maurer<br>Alexandros Polymeris<br>Marios Psychogios<br>Sebastian Thilemann<br>Christopher Traenka<br>Ioannes Tsogkas<br>Benjamin Wagner<br>Annaelle Zietz
Inselspital, University Hospital Bern	Marcel Arnold<br>Urs Fischer<br>Martina Goeldlin<br>Jan Gralla<br>Mirjam Heldner<br>Simon Jung<br>Johannes Kaesmacher<br>Basel Mamaari<br>Thomas Meinel<br>Pasquale Mordasini<br>Madlaine Mueller<br>Hakan Sarykaya<br>David Seiffge<br>Bernhard Siepen<br>Jan Vynkier
University Hospital Geneva (HUG)	Emmanuel Carrera<br>Nicolae Sanda<br>Bernardo Escribano<br>Iman Boukrid<br>Paolo Machi<br>Gianmarco Bernava<br>Andrea Rosi<br>Jeremy Hofmeister

University Hospital Lausanne (CHUV)	Ashraf Eskandari<br>Patrick Michel<br>Vasiliki Pantazou<br>Davide Strambo
Cantonal Hospital Lucerne (LUKS)	Manuel Bolognese
Neurocenter of Southern Switzerland, Lugano (EOC)	Carlo Cereda
Cantonal hospital of St. Gallen (KSSG)	Gian Marco De Marchis<br>Tolga Dittrich<br>Georg Kaegi<br>Jochen Vehoff
University Hospital Zuerich (USZ)	Mira Katan<br>Andreas Luft<br>Achim Mueller<br>Susanne Wegener
Hirslanden Zürich	Nils Peters
**Stroke Units (alphabetical order)**	
Cantonal hospital of Baden	Alexander Tarnutzer
Spietalzentrum Biel (SZB)	Stephan Salmen
Cantonal hospital Graubuenden Chur	Sylvan Albert<br>Rolf Sturzenegger
HFR Fribourg – Hôpital Cantonal, Fribourg	Andrea Humm<br>Friedrich Medlin<br>David Cuendet<br>Jean-Marie Annoni<br>Ettore Accolla<br>Sandrine Foucras
Cantonal Hospital Fribourg	Andrea Humm<br>Friedrich Medlin
Spital Sarganserland Grabs	Christian Berger
Cantonal hospital Münsterlingen	Markus Baumgärtner
Cantonal hospital Neuchâtel	Gabriele Brodo<br>Maria Cordier<br>Vaiva Jurgutiene<br>Peter Kelemen<br>Philippe Olivier<br>Susanne Renaud
GHOL Nyon	Loraine Fisch<br>Julien Niederhauser<br>Guillermo Toledo Sotomayor
Hôpital du Valais Sion	Christophe Bonvin
Bürgerspital Solothurn	Michael Schaefer
Stadtspital Waid und Triemli	Marie-Luise Mono
Cantonal hospital Winterthur	Biljana Rodic

Supplemental Methods

Data S1. List of all baseline and follow-up variables extracted from the SSR

For the analyses, we used the following prospectively collected variables: demographics (age, age group [18-45, 46-55], sex), body mass index (BMI), past medical history and cardiovascular risk factors (hypertension, hyperlipidemia, diabetes mellitus, atrial fibrillation/flutter, active smoking, previous transient ischemic attack [TIA] and intracranial hemorrhage, coronary heart disease, prosthetic heart valves, documented low ejection fraction [<35%]), the National Institutes of Health Stroke Scale (NIHSS) on admission, baseline laboratory studies (glucose, cholesterol, ldl-cholesterol, creatinine), transthoracic- and transesophageal echocardiogram (TTE, TEE), acute treatment (start antiplatelet therapy, start anticoagulants [therapeutic dose], intravenous thrombolysis with rtPA [IVT], intra-arterial treatment [IAT]), NIHSS 24h after admission, functional outcome at follow-up (mRS, mRS 0-2 vs. 3-6, mRS 0-1 vs. 2-6, NIHSS) as well as the rate of recurrent strokes and deaths until follow up.

Data S2. Measures to improve data integrity

A keyword search within patient descriptions identified as ‘other determined stroke etiology’ was conducted: Cases noting ‘embolic stroke of unknown etiology (ESUS)’ were reclassified as ‘undetermined etiology’. Cases reporting functional neurologic symptoms or stroke mimics were excluded from the study. Additionally, cases classified as “other determined etiology” described as having hypertensive or microangiopathic origins were reclassified as small vessel disease, those mentioning large artery dissection were reclassified as cervical artery dissection, and those undergoing PFO closure were categorized under PFO.

Patients in the undetermined etiology category who had atrial fibrillation or mechanical prosthetic valves – defined as high-risk sources of cardioembolism in TOAST – were reclassified as cardioembolism. Those with biological prosthetic valves or a documented low cardiac ejection fraction (<35%) – considered medium-risk sources of cardiac embolism – were excluded from analysis.

Data S3. Information on the R packages used

We used the ‘gtsummary’ package for descriptive tables and exploratory group comparisons and the ‘tidycmprsk’ and ‘ggsurvfit’ packages for competing risk analysis and cumulative incidence plots.

Table S1: Patient characteristics in patients with determined vs. undetermined stroke etiology

Characteristic	N	**Overall**, N = 3,995*	**Determined etiology**, N = 3,132*	**Undetermined etiology**, N = 863*	p-value†
**age**	3,995	48.2 (41.5, 52.1)	48.1 (41.2, 52.0)	48.6 (42.9, 52.4)	0.018
**age group**	3,995				0.003
18-45		943 (24%)	773 (25%)	170 (20%)	
46-55		3,052 (76%)	2,359 (75%)	693 (80%)	
**sex**	3,992	2,627 (66%)	2,080 (66%)	547 (63%)	0.10
**BMI**	3,202	25.7 (23.1, 29.3)	25.7 (23.0, 29.1)	26.2 (23.5, 29.7)	0.004
**BMI ≥ 25**	3,202	1,830 (57%)	1,395 (56%)	435 (62%)	0.004
**BMI ≥ 30**	3,202	699 (22%)	528 (21%)	171 (24%)	0.074
**hypertension**	3,995	1,485 (37%)	1,169 (37%)	316 (37%)	0.7
**hyperlipidemia**	3,988	2,206 (55%)	1,694 (54%)	512 (59%)	0.007
**diabetes**	3,995	416 (10%)	341 (11%)	75 (8.7%)	0.071
**smoking**	3,987	1,522 (38%)	1,152 (37%)	370 (43%)	0.001
**number of VRF**	3,995	1.0 (1.0, 2.0)	1.0 (1.0, 2.0)	2.0 (1.0, 2.0)	0.007
**≥ 1 VRF**	3,995	3,131 (78%)	2,415 (77%)	716 (83%)	<0.001
**≥ 2 VRF**	3,995	1,974 (49%)	1,522 (49%)	452 (52%)	0.054
**≥ 3 VRF**	3,995	894 (22%)	691 (22%)	203 (24%)	0.4
**atrial fibrillation/flutter**	3,995	182 (4.6%)	182 (5.8%)	0 (0%)	<0.001
**coronary heart disease**	3,988	188 (4.7%)	158 (5.1%)	30 (3.5%)	0.066
**prosthetic valves**	3,990				<0.001
None		3,901 (98%)	3,040 (97%)	861 (100%)	
Mechanical		68 (1.7%)	68 (2.2%)	0 (0%)	
Biological		21 (0.5%)	20 (0.6%)	1 (0.1%)	
**documented low ejection fraction (<35%)**	3,118	72 (2.3%)	69 (2.9%)	3 (0.4%)	<0.001
**prior TIA**	3,995	110 (2.8%)	90 (2.9%)	20 (2.3%)	0.4
**prior intracerebral hemorrhage**	3,994	31 (0.8%)	20 (0.6%)	11 (1.3%)	0.10
**cholesterol total [mmol/l]**	3,187	4.9 (4.1, 5.7)	4.8 (4.0, 5.7)	5.0 (4.2, 5.7)	0.008
**cholesterol LDL [mmol/l]**	3,144	3.0 (2.3, 3.7)	3.0 (2.3, 3.7)	3.1 (2.4, 3.7)	0.016
**creatinine [mmol/l]**	3,612	75.0 (64.0, 87.0)	75.0 (64.0, 86.0)	76.0 (65.0, 88.0)	0.2
**initial NIHSS**	3,951	2.0 (1.0, 7.0)	2.0 (1.0, 7.0)	2.0 (1.0, 6.0)	>0.9
**TTE**	3,559	2,384 (67%)	1,833 (66%)	551 (72%)	0.001
**TEE**	3,521	1,355 (38%)	986 (36%)	369 (48%)	<0.001
**start antiplatelet therapy**	3,991	2,205 (55%)	1,691 (54%)	514 (60%)	0.005
**start anticoagulants**	3,990	300 (7.5%)	275 (8.8%)	25 (2.9%)	<0.001
**intravenous thrombolysis**	3,847	1,073 (28%)	817 (27%)	256 (31%)	0.046
**intra-arterial treatment**	3,849	769 (20%)	617 (20%)	152 (18%)	0.2

Characteristic	N	**Overall**, N = 3,995*	**Determined etiology**, N = 3,132*	**Undetermined etiology**, N = 863*	**p-value**†
**NIHSS 24h after admission**	3,571	1.0 (0.0, 4.0)	1.0 (0.0, 4.0)	1.0 (0.0, 3.0)	0.060
**mRS at follow-up**	3,445				<0.001
0		1,288 (37%)	966 (36%)	322 (43%)	
1		1,022 (30%)	820 (30%)	202 (27%)	
2		699 (20%)	552 (20%)	147 (20%)	
3		207 (6.0%)	165 (6.1%)	42 (5.6%)	
4		121 (3.5%)	93 (3.4%)	28 (3.7%)	
5		15 (0.4%)	13 (0.5%)	2 (0.3%)	
6		93 (2.7%)	88 (3.3%)	5 (0.7%)	
**mRS at follow-up 0-2 vs. 3-6**	3,445				0.033
0-2		3,009 (87%)	2,338 (87%)	671 (90%)	
3-6		436 (13%)	359 (13%)	77 (10%)	
**mRS at follow-up 0-1 vs. 2-6**	3,445				0.054
0-1		2,310 (67%)	1,786 (66%)	524 (70%)	
2-6		1,135 (33%)	911 (34%)	224 (30%)	
**recurrent stroke until follow up**	3,394	76 (2.2%)	55 (2.1%)	21 (2.8%)	0.3
**death until follow up**	3,400	40 (1.2%)	39 (1.5%)	1 (0.1%)	0.005

BMI: body mass index; VRF: vascular risk factors; TIA: transient ischemic attack; LDL: low density lipoprotein; NIHSS: National Institutes of Health Stroke Scale; TTE: transthoracic echocardiogram; TEE: transesophageal echocardiogram; mRS: modified Rankin Scale

*Median (IQR) or Frequency (%)

†Kruskal-Wallis rank sum test; Pearson's Chi-squared test

Table S2: Patient characteristics in patients with determined vs. undetermined stroke etiology grouped by age groups 18-45 and 46-55

	18-45				46-55			
**Characteristic**	**N**	**Determined etiology**, N = 773*	**Undetermined etiology**, N = 170*	**p-value**†	**N**	**Determined etiology**, N = 2,359*	**Undetermined etiology**, N = 693*	**p-value**†
**age**	943	34.7 (29.6, 38.4)	34.6 (30.3, 38.3)	0.7	3,052	50.3 (46.7, 52.9)	50.3 (46.7, 53.0)	0.9
**sex**	941	425 (55%)	88 (52%)	0.5	3,051	1,655 (70%)	459 (66%)	0.053
**etiology**	943			<0.001	3,052			<0.001
Cervical artery dissection		222 (29%)				409 (17%)		
PFO		227 (29%)				380 (16%)		
Cardiac embolism		101 (13%)				446 (19%)		
Other determined etiology		124 (16%)				258 (11%)		
Small vessel disease		32 (4.1%)				338 (14%)		
Large artery atherosclerosis		26 (3.4%)				344 (15%)		
More than one possible etiology		41 (5.3%)				184 (7.8%)		
**BMI**	785	24.6 (21.7, 27.7)	25.2 (22.7, 29.0)	0.033	2,417	25.9 (23.4, 29.4)	26.2 (23.7, 30.0)	0.070
**BMI ≥ 25**	785	285 (45%)	79 (54%)	0.038	2,417	1,110 (60%)	356 (64%)	0.081
**BMI ≥ 30**	785	100 (16%)	31 (21%)	0.12	2,417	428 (23%)	140 (25%)	0.3
**hypertension**	943	116 (15%)	21 (12%)	0.4	3,052	1,053 (45%)	295 (43%)	0.4
**hyperlipidemia**	942	268 (35%)	78 (46%)	0.008	3,046	1,426 (61%)	434 (63%)	0.3
**diabetes**	943	35 (4.5%)	5 (2.9%)	0.5	3,052	306 (13%)	70 (10%)	0.051
**smoking**	940	255 (33%)	71 (42%)	0.034	3,047	897 (38%)	299 (43%)	0.016
**number of VRF**	943	1.0 (0.0, 2.0)	1.0 (0.0, 2.0)	0.011	3,052	2.0 (1.0, 3.0)	2.0 (1.0, 3.0)	0.4
**≥ 1 VRF**	943	480 (62%)	120 (71%)	0.046	3,052	1,935 (82%)	596 (86%)	0.017
**≥ 2 VRF**	943	205 (27%)	61 (36%)	0.018	3,052	1,317 (56%)	391 (56%)	0.8
**≥ 3 VRF**	943	67 (8.7%)	18 (11%)	0.5	3,052	624 (26%)	185 (27%)	>0.9
**atrial fibrillation/flutter**	943	15 (1.9%)	0 (0%)	0.14	3,052	167 (7.1%)	0 (0%)	<0.001
**coronary heart disease**	941	14 (1.8%)	1 (0.6%)	0.4	3,047	144 (6.1%)	29 (4.2%)	0.067
**prosthetic valves**	943			0.15	3,047			<0.001
None		756 (98%)	170 (100%)			2,284 (97%)	691 (100%)	
Mechanical		11 (1.4%)	0 (0%)			57 (2.4%)	0 (0%)	
Biological		6 (0.8%)	0 (0%)			14 (0.6%)	1 (0.1%)	

		18-45				46-55		
**Characteristic**	**N**	**Determined etiology**, N = 773*	**Undetermined etiology**, N = 170*	**p-value**†	**N**	**Determined etiology**, N = 2,359*	**Undetermined etiology**, N = 693*	**p-value**†
**documented low ejection fraction (<35%)**	726	13 (2.2%)	0 (0%)	0.2	2,392	56 (3.1%)	3 (0.5%)	0.001
**prior TIA**	943	20 (2.6%)	1 (0.6%)	0.2	3,052	70 (3.0%)	19 (2.7%)	0.9
**prior intracerebral hemorrhage**	943	5 (0.6%)	0 (0%)	0.6	3,051	15 (0.6%)	11 (1.6%)	0.031
**cholesterol total [mmol/l]**	714	4.4 (3.8, 5.1)	4.7 (4.1, 5.4)	0.027	2,473	4.9 (4.2, 5.8)	5.1 (4.3, 5.8)	0.2
**cholesterol LDL [mmol/l]**	705	2.6 (2.0, 3.2)	2.9 (2.2, 3.5)	0.011	2,439	3.1 (2.3, 3.9)	3.2 (2.4, 3.7)	0.3
**creatinine [mmol/l]**	830	72.0 (62.0, 84.0)	70.0 (61.0, 83.0)	0.3	2,782	76.0 (65.0, 87.0)	77.0 (66.0, 90.0)	0.083
**initial NIHSS**	929	2.0 (0.0, 6.0)	2.0 (1.0, 7.0)	0.12	3,022	3.0 (1.0, 7.0)	2.0 (1.0, 6.0)	0.3
**TTE**	849	438 (63%)	102 (68%)	0.3	2,710	1,395 (67%)	449 (73%)	0.004
**TEE**	839	307 (44%)	90 (60%)	<0.001	2,682	679 (33%)	279 (46%)	<0.001
**start antiplatelet therapy**	943	399 (52%)	96 (56%)	0.3	3,048	1,292 (55%)	418 (60%)	0.012
**start anticoagulants**	942	72 (9.3%)	3 (1.8%)	0.002	3,048	203 (8.6%)	22 (3.2%)	<0.001
**intravenous thrombolysis**	906	211 (28%)	53 (32%)	0.4	2,941	606 (27%)	203 (30%)	0.069
**intra-arterial treatment**	908	161 (22%)	34 (21%)	0.8	2,941	456 (20%)	118 (18%)	0.2
**NIHSS 24h after admission**	847	1.0 (0.0, 3.0)	1.0 (0.0, 3.0)	0.5	2,724	1.0 (0.0, 4.0)	1.0 (0.0, 3.0)	0.009
**mRS at follow-up**	818			0.6	2,627			<0.001
0		264 (40%)	70 (45%)			702 (34%)	252 (43%)	
1		213 (32%)	40 (26%)			607 (30%)	162 (27%)	
2		130 (20%)	35 (22%)			422 (21%)	112 (19%)	
3		27 (4.1%)	7 (4.5%)			138 (6.8%)	35 (5.9%)	
4		16 (2.4%)	3 (1.9%)			77 (3.8%)	25 (4.2%)	
5		1 (0.2%)	0 (0%)			12 (0.6%)	2 (0.3%)	
6		11 (1.7%)	1 (0.6%)			77 (3.8%)	4 (0.7%)	
**mRS at follow-up 0-2 vs. 3-6**	818			0.7	2,627			0.023
0-2		607 (92%)	145 (93%)			1,731 (85%)	526 (89%)	
3-6		55 (8.3%)	11 (7.1%)			304 (15%)	66 (11%)	
**mRS at follow-up 0-1 vs. 2-6**	818			0.8	2,627			0.013
0-1		477 (72%)	110 (71%)			1,309 (64%)	414 (70%)	
2-6		185 (28%)	46 (29%)			726 (36%)	178 (30%)	

		18-45				46-55		
**Characteristic**	**N**	**Determined etiology**, N = 773*	**Undetermined etiology**, N = 170*	**p-value**†	**N**	**Determined etiology**, N = 2,359*	**Undetermined etiology**, N = 693*	**p-value**†
**recurrent stroke until follow up**	806	12 (1.8%)	7 (4.5%)	0.10	2,588	43 (2.2%)	14 (2.4%)	0.9
**death until follow up**	807	0 (0%)	1 (0.6%)	0.4	2,593	39 (2.0%)	0 (0%)	0.001

PFO: patent foramen ovale; BMI: body mass index; VRF: vascular risk factors; TIA: transient ischemic attack; LDL: low density lipoprotein; NIHSS: National Institutes of Health Stroke Scale; TTE: transthoracic echocardiogram; TEE: transesophageal echocardiogram; mRS: modified Rankin Scale

*Median (IQR) or Frequency (%)

†Kruskal-Wallis rank sum test; Pearson's Chi-squared test

Table S3: Logistic regression model predicting favorable functional outcomes (mRS 0-2) at 90-day follow-up

Characteristic	OR*	95% CI*	p-value
age	0.95	0.93, 0.96	<0.001
etiology			
Determined etiology	—	—	
Undetermined etiology	1.25	0.83, 1.94	0.3
age * etiology			
age * Undetermined etiology	1.04	1.00, 1.08	0.027
etiology * sex			
Determined etiology * Male	1.08	0.84, 1.37	0.5
Undetermined etiology * Male	1.12	0.68, 1.81	0.7

*OR = Odds Ratio, CI = Confidence Interval

Null deviance = 2,612; Null df = 3,441; Log-likelihood = -1,281; AIC = 2,574; BIC = 2,611; Deviance = 2,562; Residual df = 3,436; No. Obs. = 3,442

This table displays results from a logistic regression model predicting 90-day favorable functional outcomes (mRS 0-2), factoring in dichotomized stroke etiologies (determined (reference) vs. undetermined etiology), mean centered age, and sex (female as reference) as well as the interactions between age and etiology and sex and etiology. It details odds ratios, confidence intervals, and p-values for these variables, using Maximum Likelihood estimation.

Table S4: Logistic regression model predicting favorable functional outcomes (mRS 0-2) in the 18-45 age group at 90-day follow-up

Characteristic	OR*	95% CI*	p-value
age	0.95	0.89, 1.00	0.049
etiology			
Determined etiology	—	—	
Undetermined etiology	1.55	0.61, 4.81	0.4
age * etiology			
age * Undetermined etiology	0.97	0.82, 1.11	0.7
etiology * sex			
Determined etiology * Male	1.46	0.83, 2.58	0.2
Undetermined etiology * Male	0.98	0.27, 3.46	>0.9

*OR = Odds Ratio, CI = Confidence Interval

Null deviance = 454; Null df = 815; Log-likelihood = -223; AIC = 458; BIC = 486; Deviance = 446; Residual df = 810; No. Obs. = 816

This table displays results from a logistic regression model predicting 90-day favorable functional outcomes (mRS 0-2) in the 18-45 age group, factoring in dichotomized stroke etiologies (determined (reference) vs. undetermined etiology), mean centered age, and sex (female as reference) as well as the interactions between age and etiology and sex and etiology. It details odds ratios, confidence intervals, and p-values for these variables, using Maximum Likelihood estimation.

Table S5: Logistic regression model predicting favorable functional outcomes (mRS 0-2) in the 46-55 age group at 90-day follow-up

Characteristic	OR[*]	95% CI[*]	p-value
age	0.92	0.89, 0.95	<0.001
etiology			
Determined etiology	—	—	
Undetermined etiology	1.27	0.80, 2.11	0.3
age * etiology			
age * Undetermined etiology	1.15	1.07, 1.24	<0.001
etiology * sex			
Determined etiology * Male	1.01	0.77, 1.32	>0.9
Undetermined etiology * Male	1.17	0.68, 1.97	0.6

[*]OR = Odds Ratio, CI = Confidence Interval

Null deviance = 2,135; Null df = 2,625; Log-likelihood = -1,052; AIC = 2,115; BIC = 2,150; Deviance = 2,103; Residual df = 2,620; No. Obs. = 2,626

This table displays results from a logistic regression model predicting 90-day favorable functional outcomes (mRS 0-2) in the 46-55 age group, factoring in dichotomized stroke etiologies (determined (reference) vs. undetermined etiology), mean centered age, and sex (female as reference) as well as the interactions between age and etiology and sex and etiology. It details odds ratios, confidence intervals, and p-values for these variables, using Maximum Likelihood estimation.

Table S6: Logistic regression model predicting excellent functional outcomes (mRS 0-1) at 90-day follow-up

Characteristic	OR*	95% CI*	p-value
age	0.97	0.96, 0.98	<0.001
etiology			
Determined etiology	—	—	
Undetermined etiology	1.16	0.87, 1.55	0.3
age * etiology			
age * Undetermined etiology	1.03	1.00, 1.05	0.024
etiology * sex			
Determined etiology * Male	1.18	0.99, 1.40	0.065
Undetermined etiology * Male	1.23	0.89, 1.71	0.2

*OR = Odds Ratio, CI = Confidence Interval

Null deviance = 4,362; Null df = 3,441; Log-likelihood = -2,164; AIC = 4,341; BIC = 4,377; Deviance = 4,329; Residual df = 3,436; No. Obs. = 3,442

This table displays results from a logistic regression model predicting 90-day excellent functional outcomes (mRS 0-1), factoring in dichotomized stroke etiologies (determined (reference) vs. undetermined etiology), mean centered age, and sex (female as reference) as well as the interactions between age and etiology and sex and etiology. It details odds ratios, confidence intervals, and p-values for these variables, using Maximum Likelihood estimation.

Table S7: Logistic regression model predicting excellent functional outcomes (mRS 0-1) in the 18-45 age group at 90-day follow-up

Characteristic	OR*	95% CI*	p-value
age	0.94	0.91, 0.97	<0.001
etiology			
Determined etiology	—	—	
Undetermined etiology	1.01	0.59, 1.77	>0.9
age * etiology			
age * Undetermined etiology	1.08	1.01, 1.16	0.028
etiology * sex			
Determined etiology * Male	1.33	0.94, 1.88	0.11
Undetermined etiology * Male	1.05	0.53, 2.11	0.9

*OR = Odds Ratio, CI = Confidence Interval

Null deviance = 969; Null df = 815; Log-likelihood = -476; AIC = 963; BIC = 991; Deviance = 951; Residual df = 810; No. Obs. = 816

This table displays results from a logistic regression model predicting 90-day excellent functional outcomes (mRS 0-1) in the 18-45 age group, factoring in dichotomized stroke etiologies (determined (reference) vs. undetermined etiology), mean centered age, and sex (female as reference) as well as the interactions between age and etiology and sex and etiology. It details odds ratios, confidence intervals, and p-values for these variables, using Maximum Likelihood estimation.

Table S8: Logistic regression model predicting excellent functional outcomes (mRS 0-1) in the 46-55 age group at 90-day follow-up

Characteristic	OR*	95% CI*	p-value
age	0.99	0.96, 1.01	0.2
etiology			
Determined etiology	—	—	
Undetermined etiology	1.19	0.85, 1.67	0.3
age * etiology			
age * Undetermined etiology	1.02	0.97, 1.07	0.5
etiology * sex			
Determined etiology * Male	1.13	0.92, 1.37	0.2
Undetermined etiology * Male	1.29	0.89, 1.86	0.2

*OR = Odds Ratio, CI = Confidence Interval

Null deviance = 3,381; Null df = 2,625; Log-likelihood = -1,685; AIC = 3,382; BIC = 3,418; Deviance = 3,370; Residual df = 2,620; No. Obs. = 2,626

This table displays results from a logistic regression model predicting 90-day excellent functional outcomes (mRS 0-1) in the 46-55 age group, factoring in dichotomized stroke etiologies (determined (reference) vs. undetermined etiology), mean centered age, and sex (female as reference) as well as the interactions between age and etiology and sex and etiology. It details odds ratios, confidence intervals, and p-values for these variables, using Maximum Likelihood estimation.

Table S9: Logistic regression model of age, sex and stroke etiology predicting favorable functional outcomes (mRS 0-2) at 90-day follow-up

Characteristic	OR*	95% CI*	p-value
age	0.95	0.94, 0.97	<0.001
sex			
Female	—	—	
Male	1.05	0.84, 1.31	0.7
etiology			
Undetermined etiology	—	—	
Cervical artery dissection	0.55	0.39, 0.77	<0.001
PFO	2.35	1.46, 3.92	<0.001
Cardiac embolism	0.71	0.50, 1.01	0.054
Other determined etiology	0.34	0.24, 0.49	<0.001
Small vessel disease	1.78	1.08, 3.04	0.028
Large artery atherosclerosis	0.61	0.42, 0.90	0.011
More than one possible etiology	0.68	0.43, 1.09	0.10

PFO: patent foramen ovale

*OR = Odds Ratio, CI = Confidence Interval

Null deviance = 2,612; Null df = 3,441; Log-likelihood = -1,235; AIC = 2,489; BIC = 2,551; Deviance = 2,469; Residual df = 3,432; No. Obs. = 3,442

This table displays results from a logistic regression model predicting 90-day favorable functional outcomes (mRS 0-2), factoring in stroke etiologies (with undetermined etiology as the reference), mean centered age, and sex (female as reference). It details odds ratios, confidence intervals, and p-values for these variables, using Maximum Likelihood estimation.

Table S10: Logistic regression model of age, sex and stroke etiology predicting excellent functional outcomes (mRS 0-1) at 90-day follow-up

Characteristic	OR*	95% CI*	p-value
age	0.98	0.97, 0.99	<0.001
sex			
Female	—	—	
Male	1.17	1.01, 1.37	0.041
etiology			
Undetermined etiology	—	—	
Cervical artery dissection	0.55	0.43, 0.69	<0.001
PFO	1.36	1.05, 1.76	0.020
Cardiac embolism	0.89	0.69, 1.14	0.4
Other determined etiology	0.53	0.41, 0.69	<0.001
Small vessel disease	1.30	0.96, 1.76	0.10
Large artery atherosclerosis	0.71	0.53, 0.94	0.015
More than one possible etiology	1.00	0.71, 1.42	>0.9

PFO: patent foramen ovale

*OR = Odds Ratio, CI = Confidence Interval

Null deviance = 4,358; Null df = 3,438; Log-likelihood = -2,126; AIC = 4,271; BIC = 4,333; Deviance = 4,251; Residual df = 3,429; No. Obs. = 3,439

This table displays results from a logistic regression model predicting 90-day excellent functional outcomes (mRS 0-1), factoring in stroke etiologies (with undetermined etiology as the reference), mean centered age, and sex (female as reference). It details odds ratios, confidence intervals, and p-values for these variables, using Maximum Likelihood estimation.

Table S11: Fine-Gray proportional hazards models for stroke recurrence based on dichotomized stroke etiology

Characteristic	HR*	95% CI*	p-value
etiology			
Determined etiology	—	—	
Undetermined etiology	1.72	1.01, 2.94	0.046
age	0.99	0.96, 1.02	0.4
sex	1.00	0.59, 1.71	>0.9

*HR = Hazard Ratio, CI = Confidence Interval

converged = 1.00; Log-likelihood = -494; No. Obs. = 3,217; df = 3; Statistic = 4.42

Table S12: Fine-Gray proportional hazards models for stroke recurrence based on dichotomized stroke etiology in patients aged 18 to 45

Characteristic	HR*	95% CI*	p-value
etiology			
Determined etiology	—	—	
Undetermined etiology	3.24	1.15, 9.11	0.026
age	0.97	0.89, 1.07	0.6
sex	0.83	0.30, 2.32	0.7

*HR = Hazard Ratio, CI = Confidence Interval

converged = 1.00; Log-likelihood = -97.2; No. Obs. = 756; df = 3; Statistic = 5.27

Table S13: Fine-Gray proportional hazards models for stroke recurrence based on dichotomized stroke etiology in patients aged 46 to 55

Characteristic	HR*	95% CI*	p-value
etiology			
Determined etiology	—	—	
Undetermined etiology	1.37	0.73, 2.57	0.3
age	0.98	0.91, 1.04	0.5
sex	1.07	0.58, 1.98	0.8

*HR = Hazard Ratio, CI = Confidence Interval

converged = 1.00; Log-likelihood = -360; No. Obs. = 2,461; df = 3; Statistic = 1.47

Table S14: Fine-Gray proportional hazards models for stroke recurrence based on stroke etiology

Characteristic	HR*	95% CI*	p-value
etiology			
Undetermined etiology	—	—	
Cervical artery dissection	0.46	0.19, 1.09	0.078
PFO	0.12	0.03, 0.54	0.005
Cardiac embolism	0.65	0.29, 1.46	0.3
Other determined etiology	0.97	0.44, 2.13	>0.9
Small vessel disease	0.49	0.17, 1.43	0.2
Large artery atherosclerosis	1.01	0.45, 2.31	>0.9
More than one possible etiology	0.95	0.36, 2.51	>0.9
age	0.98	0.95, 1.01	0.2
sex	1.00	0.59, 1.71	>0.9

PFO: patent foramen ovale

*HR = Hazard Ratio, CI = Confidence Interval

converged = 1.00; Log-likelihood = -487; No. Obs. = 3,217; df = 9; Statistic = 18.6

Results from a Fine-Gray proportional hazards model evaluating 90-day stroke recurrence, considering death as a competing risk. This model adjusts for age and sex, with undetermined etiology as the reference group for stroke etiology comparison.

Figure S1: Vascular risk factors in patients with undetermined and determined etiologies across age categories. (a) Percentage distribution of hypertension, hyperlipidemia, diabetes, smoking, and obesity. (b) Boxplot distribution of the number of cardiovascular risk factors.

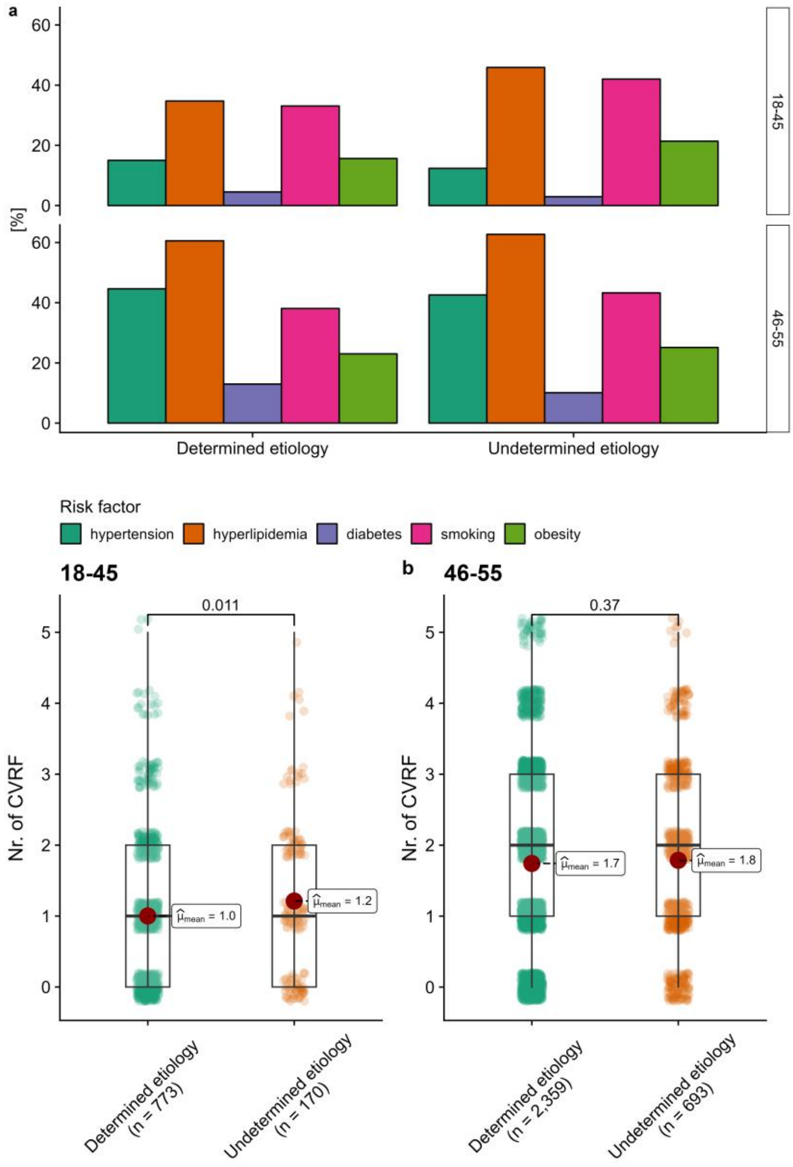

Figure S2: Coefficient plot of a logistic regression model predicting favorable functional outcomes (mRS 0-2) at 90-day follow-up

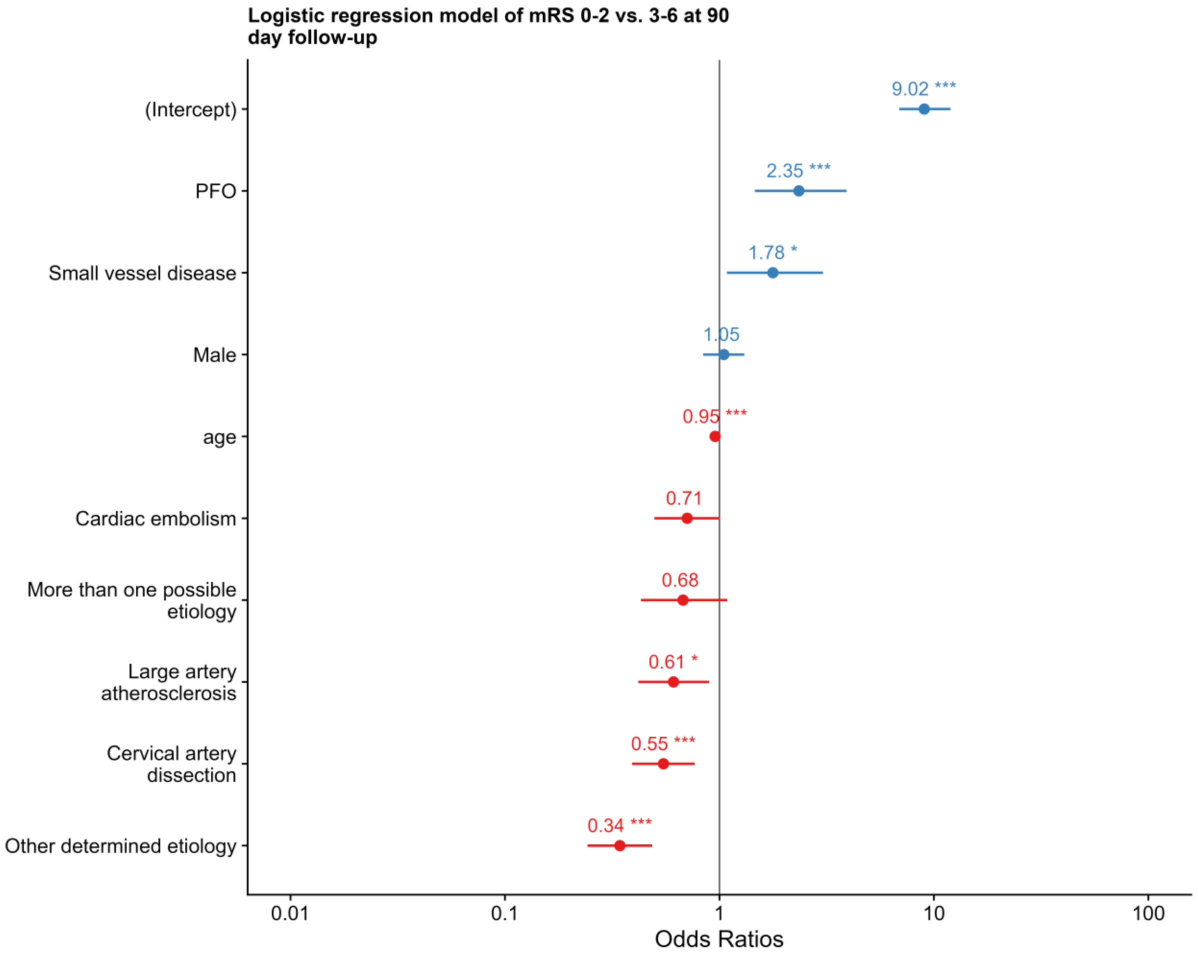

PFO: patent foramen ovale

Coefficient plot from a logistic regression model showing odds ratios for predicting favorable functional outcomes (mRS 0-2) at 90 days. Variables are stroke etiology (reference = undetermined etiology), mean centered age, and sex (reference = female), with asterisks denoting significance levels (*p < 0.05, **p < 0.01, ***p < 0.001)

Figure S3: Coefficient plot of a logistic regression model predicting excellent functional outcomes (mRS 0-1) at 90-day follow-up

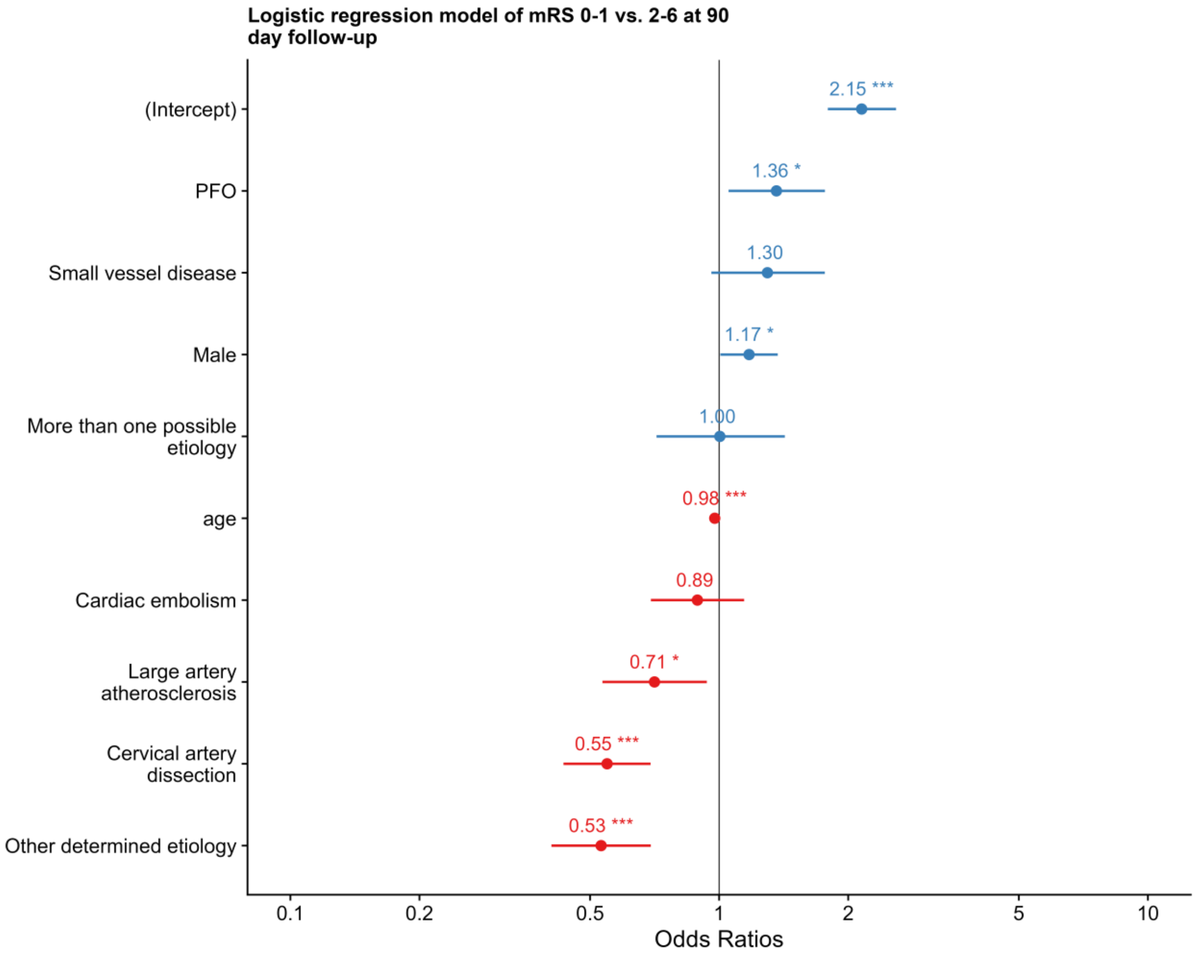

PFO: patent foramen ovale

Coefficient plot from a logistic regression model showing odds ratios for predicting excellent functional outcomes (mRS 0-1) at 90 days. Variables are stroke etiology (reference = undetermined etiology), mean centered age, and sex (reference = female), with asterisks denoting significance levels (*p < 0.05, **p < 0.01, ***p < 0.001)
